# GATA3 is a sensitive marker for primary genital extramammary paget disease: an immunohistochemical study of 72 cases with comparison to gross cystic disease fluid protein 15

**DOI:** 10.1186/s13000-017-0638-z

**Published:** 2017-07-10

**Authors:** Ming Zhao, Lixin Zhou, Li Sun, Yan Song, Yunquan Guo, Xun Zhang, Feng Zhao, Peng Wang, Junqiu Yue, Dongfeng Niu, Zhongwu Li, Xiaozheng Huang, Qiang Kang, Lin Jia, Jinping Lai, Dengfeng Cao

**Affiliations:** 1 Department of Pathology, Zhejiang Provincial People’s Hospital, Hangzhou, China; 2 Department of Pathology, Key Laboratory of Carcinogenesis and Translational Research (Ministry of Education), Peking University Cancer Hospital (Beijing Cancer Hospital), Beijing, China; 3 Department of Pathology, Cancer Hospital of Chinese Academy of Medical Sciences, Beijing, China; 4 Department of Pathology, Xinjiang Medical University Affiliated Tumor Hospital, Urumqi, China; 5 Department of Pathology, Beijing Ditan Hospital, Beijing, China; 6 Department of Pathology, Hubei Cancer Hospital, Wuhan, China; 7 Department of Pathology, Saint Louis University School of Medicine, Saint Louis, MO USA; 8 Department of Pathology and Immunology, Washington University School of Medicine, 660 S South Euclid Avenue Campus Box 8118, Saint Louis, MO 63110 USA

**Keywords:** GATA3, GCDFP15, Extramammary Paget disease, Immunohistochemical marker

## Abstract

**Background:**

GATA-binding protein 3 (GATA3) has been identified as a sensitive marker for breast carcinoma but its sensitivity in primary genital extramammary Paget diseases (EMPDs) has not been well studied.

**Methods:**

Here we investigated immunohistochemical expression of GATA3 in 72 primary genital EMPDs (35 from female, 37 from male; 45 with intraepithelial disease only, 26 with both intraepithelial disease and invasive adenocarcinoma including 14 also metastasis, 1 with metastatic adenocarcinoma only for study). We also compared GATA3 to gross cystic disease fluid protein 15 (GCDFP15) for their sensitivity.

**Results:**

Positive GATA3 staining was seen in all 71 (100%) intraepithelial diseases, 25/26 (96%; female 10/10, male 15/16) invasive adenocarcinomas and 14/15 (93%; female 3/3, male 11/12) metastatic adenocarcinomas, respectively. Positive GCDFP15 staining was seen in 46/71 (65%; female 28/34 or 82%, male 18/37 or 49%) intraepithelial diseases, 20/26 (77%; female 9/10, male 11/16) invasive adenocarcinomas, and 12/15 (80%; female 2/3, male 10/12) metastatic adenocarcinomas, respectively (GATA3 versus GCDFP15: *p* < 0.01 for both intraepithelial disease and invasive adenocarcinoma, *p* = 0.28 for metastatic adenocarcinoma). In positive-stained cases, GATA3 stained more tumor cells than GCDFP15 (79% versus 25% for intraepithelial disease, 71% vs 34% for invasive adenocarcinoma, 73% vs 50% for metastatic adenocarcinoma, *p* < 0.01 for all 3 components).

**Conclusions:**

Our findings indicate that GATA3 is a very sensitive marker for primary genital EMPDs and is more sensitive than GCDFP15.

## Background

Paget disease (PD) is a distinct intraepidermal adenocarcinoma with a pagetoid growth pattern. PDs are classified as mammary and extramammary subtypes according to their locations and their relationship to breast [1, 2]. Mammary PDs account for 90% of the PDs occurring on the skin of nipple/areola complex and most of them represent tumor spread to the epidermis from an underlying invasive ductal carcinoma (53–60%) or ductal carcinoma in situ (24–43%). Compared to breast PD, primary extramammary PDs (EMPDs) are relatively uncommon and their histogenesis is less clear [1, 2].

Primary EMPDS are found in areas rich in apocrine glands. The most common site of primary EMPDs is vulva followed by perianal skin, scrotum and penis, and axilla etc. [1–6]. In women, more than 80% of primary EMPDs are in the vulva [1–4, 6]. In men, approximately half of EMPDs are in the penoscrotal region [4–8]. Most primary EMPDs are intraepithelial at their initial presentation (type Ia disease) but some have both intraepithelial disease and invasive adenocarcinoma i.e. invasive EMPDs [1–11]. The invasive adenocarcinomas seen in primary EMPDs include those arising from intraepithelial EMPD (type Ib disease) and those giving rise to the intraepithelial disease (type Ic disease, underlying adenocarcinoma with subsequent epidermal involvement i.e. Paget disease as manifestation of an underlying adenocarcinoma) [3]. Among patients with invasive EMPDs (type Ib and type Ic), 20% to 40% had lymph node metastasis [4–7, 9, 11]. Up to 17% to 50% patients with invasive EMPDs also develop concurrent or subsequent distant metastasis [4, 5, 7, 9–12].

Primary EMPDs should be distinguished from secondary EMPDs given their different treatment and prognosis [3]. Secondary EMPD is usually the result of intraepithelial spread from a visceral carcinoma located elsewhere, with the gastrointestinal tract (colorectum) or urogenital tract (urinary bladder, prostate) being the most 2 common sources [1–3, 9, 13–17]. EMPDs may also pose some diagnostic challenges in metastatic sites as they morphologically may mimic other tumors such as urothelial carcinoma and breast carcinoma. This diagnostic challenge is further complicated by the fact that patients with EMPDs have an increased risk of developing secondary primary tumors in which breast carcinoma, colorectal adenocarcinoma and urothelial carcinoma are among the most common ones [4–6, 9, 14–17].

Given the overlapping morphologic features between primary EMPDs and secondary ones, and between metastatic EMPDs and their mimics in metastatic sites, immunohistochemical markers are often needed to facilitate the correct diagnosis. Several immunohistochemical markers, including cytokeratin 7, carcinoembryonic antigen, androgen receptor and c-erbB2 (HER2), have been used for diagnosing primary EMPDs, however, their specificity is relatively low [18–20] and therefore limited their diagnostic utility in metastatic setting. Gross cystic duct fluid protein 15 (GCDFP15, also known as BRST-2) shows relatively high specificity for EMPDs but its sensitivity was only 60% to 85% and in many cases the staining was focal [21–26]. Primary EMPD is analogous to breast Paget disease. Recently a transcription factor GATA-binding protein 3 (GATA3) has been identified as a very sensitive marker for breast carcinoma, both in both primary and metastatic sites [27–31]. GATA3 was also reported to be highly expressed in apocrine glands and adnexal tumors [30]. Apocrine gland has been proposed as the origin of primary EPMDs according to one theory [1, 2, 21]. These findings suggest that GATA3 might be a sensitive marker for primary EMPDs. In the literature, there was only one recent report of GATA3 in 11 vulvar primary EMPDs [32].

In this study, using immunohistochemical staining we investigated the expression of GATA3 in a large series of 72 primary EMPDs (45 with intraepithelial disease only, 26 with both intraepithelial disease and invasive adenocarcinoma including 14 also with lymph node metastasis, 1 with metastatic adenocarcinoma only for study) in male and female genital regions to explore the potential diagnostic utility of GATA3 in these tumors. We also compared GATA3 to GCDFP15 in these tumors for their sensitivity.

## Methods

### Materials

The surgical pathology archives of the authors’ hospitals were searched for primary EMPDs in male and female genital regions. A total of 72 surgically resected cases with confirmed diagnosis of primary EMPDs in the genital region were included for this study: 35 from female and 37 from male patients. All 35 female cases were from vulva, including 24 with intraepithelial disease only (type Ia disease), 10 with both intraepithelial disease and invasive adenocarcinoma (5 type Ib, 5 type Ic, 2/5 type Ic cases with metastatic adenocarcinoma in nodes) and 1 with only metastatic node disease for study (history of primary vulvar EMPD). No breast tissue or mammary-like gland was present in the adjacent vulva tissue in any of these female cases. The 37 male cases included 3 from penis, 1 from perineum, and 33 from the scrotum. Twenty-one (21 or 57%) male cases were intraepithelial diseases, 16 (43%) cases had both intraepithelial disease and invasive adenocarcinoma (14 type Ib, 2 type Ic; 12/16 also with nodal metastasis including 11/14 type Ib and 1/2 type Ic).

### Immunohistochemical staining

One to two formalin-fixed, paraffin-embedded full tissue blocks from each case were retrieved to generate 4 um unstained slides for immunohistochemical staining on a Ventana Benchmark-XT automated stainer using the Ventana ultraView DAB detection kit. The antibody to GATA3 is a mouse monoclonal antibody (clone L50–823, prediluted, Biocare, Concord, CA 94520). The antibody to GCDFP15 was a rabbit monoclonal antibody (clone EP95, prediluted, Rocklin, CA 95677). The automatic immunohistochemical reaction was performed with Ventana Cell Conditioning Solution 1 (CC1) at pH 6.0. The primary antibody (antibody to GATA3, antibody to GCDFP15) was incubated at 37 degrees for 24 min. Positive control (breast ductal carcinoma as positive control) and negative control (incubation with secondary antibody only) were included for each run of immunostains. Only nuclear staining was considered positive for GATA3. The staining pattern for GCDFP15 is cytoplasmic. The percentage of tumor cells labeled was semi-quantitatively scored as 0 (<1% tumor cell staining), 1+ (1–25%), 2+ (26–50%), 3+ (51–75%), and 4+ (76–100%).

### Statistical analysis

The Fisher exact test was used to compare the staining pattern for GATA3 with GCDFP15, and paired *t*-test was used to compare the mean percentage of tumor cells stained with GATA3 with GCDFP15 in the intraepithelial component, invasive component and metastatic components of EMPDs. A *P*-value of <0.05 was considered statistically significant.

## Results

### Expression of GATA3 and GCDFP15 in female primary extramammary Paget diseases

Among the 35 vulvar EMPDs, 24 had intraepithelial disease only, 10 had both intraepithelial disease and invasive adenocarcinoma (2 also had regional lymph node metastasis) and 1 had only the metastatic adenocarcinoma in one lymph node for study (history of vulvar primary EMPD). Among the 10 cases with both intraepithelial disease and invasive adenocarcinoma, 5 were type Ib and 5 were type Ic diseases (4 apocrine carcinomas and 1 eccrine carcinoma). The staining results of GATA3 and GCDFP15 for each component of vulvar extra-mammary diseases are summarized in Table 1.

**Table 1:** Immunohistochemical staining results of GATA3 and GCDFP15 in primary vulvar extramammary Paget diseases

Disease Component	GATA3 staining^a^						GCDFP15 staining^a^						*P* value
	0	1+	2+	3+	4+	Total	0	1+	2+	3+	4+	Total	
Intraepithelial disease (*N* = 34)	0	2 (6%)	1 (3%)	3 (9%)	28 (82%)	34 (100%)	6 (8%)	12 (35%)	4 (12%)	4 (12%)	8 (24%)	28 (82%)	0.0004^#^
Type Ia (*N* = 24)		1		3	20		4	8	4	3	5		
Type Ib (*N* = 5)			1		4		1	3			1		
Type Ic (*N* = 5)		1			4		1	1		1	2		
Invasive adenocarcinoma(*N* = 10)	0	0	2 (20%)	0	8 (80%)	10 (10%)	1 (10%)	3 (30%)	0	1 (10%)	5 (50%)	9 (90%)	0.1035^#^
type Ib (*N* = 5)					5			1		1	3		
type Ic (*N* = 5)			2		3		1	2			2		
Metastatic adenocarcinoma (*N* = 3)	0	0	0	0	3 (100%)	3 (100%)	1 (33%)	1 (33%)	0	0	1 (33%)	2 (67%)	not applicable

^a^The staining is semi-quantitatively as follows: 0: <1% tumor cell staining; 1+: 1–25% tumor cells staining; 2+: 26–50% tumor cells staining; 3+: 51–75% tumor cells staining; 4+: 76–100% tumor cells staining. *NA* non-applicable due to small number

^#^ *p* value refers to comparison of staining patterns (0,1+,2+,3+,4+) not percentage of total positives

**Fig. 1:**
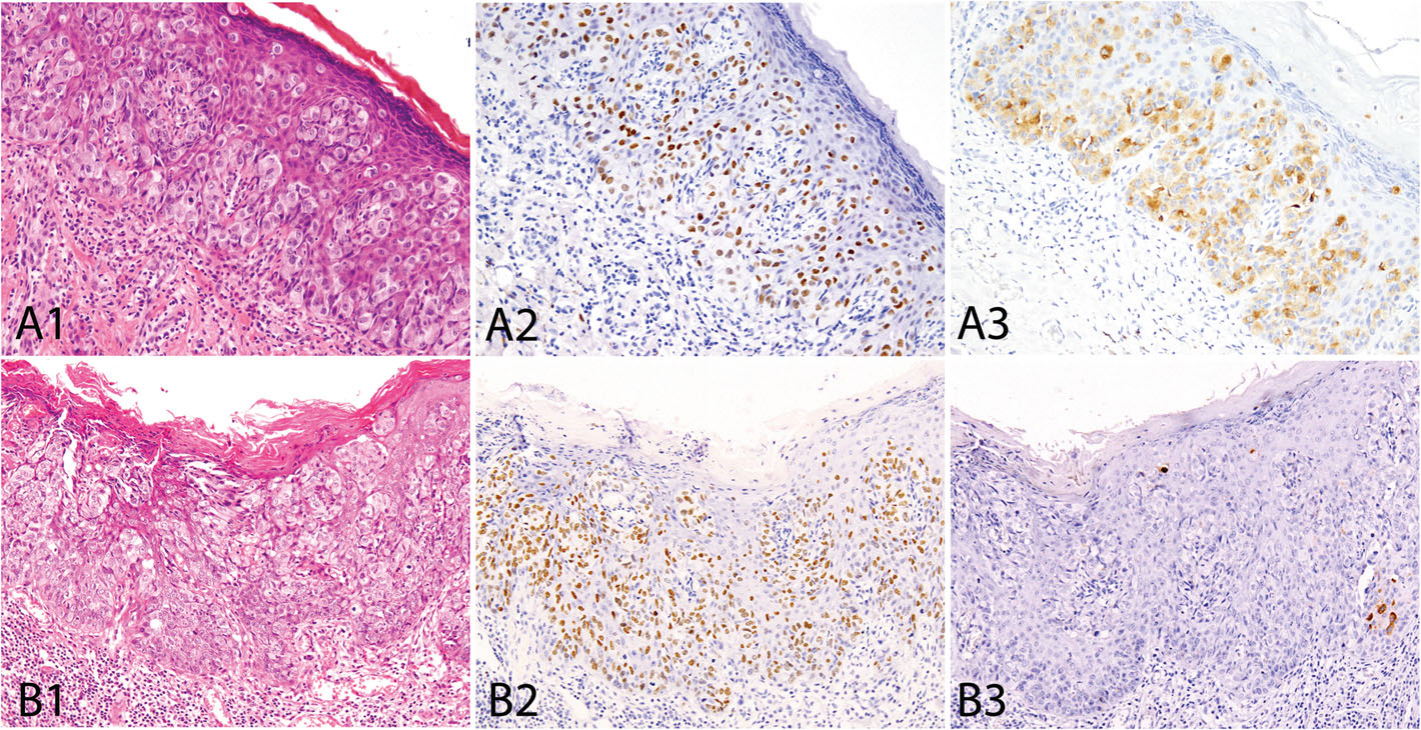
Immunohistochemical staining of GATA3 and GCDFP15 in primary extramammary Paget diseases in the genital region: all intraepithelial diseases in both genders (A1, vulva; B1, scrotum) were positive for GATA3 (A2, B2). Most of intraepithelial diseases (50% male, 82% female) were also positive for GCDFP15 (A3, B3). GCDFP15 staining is often focal (B3)

**Fig. 2:**
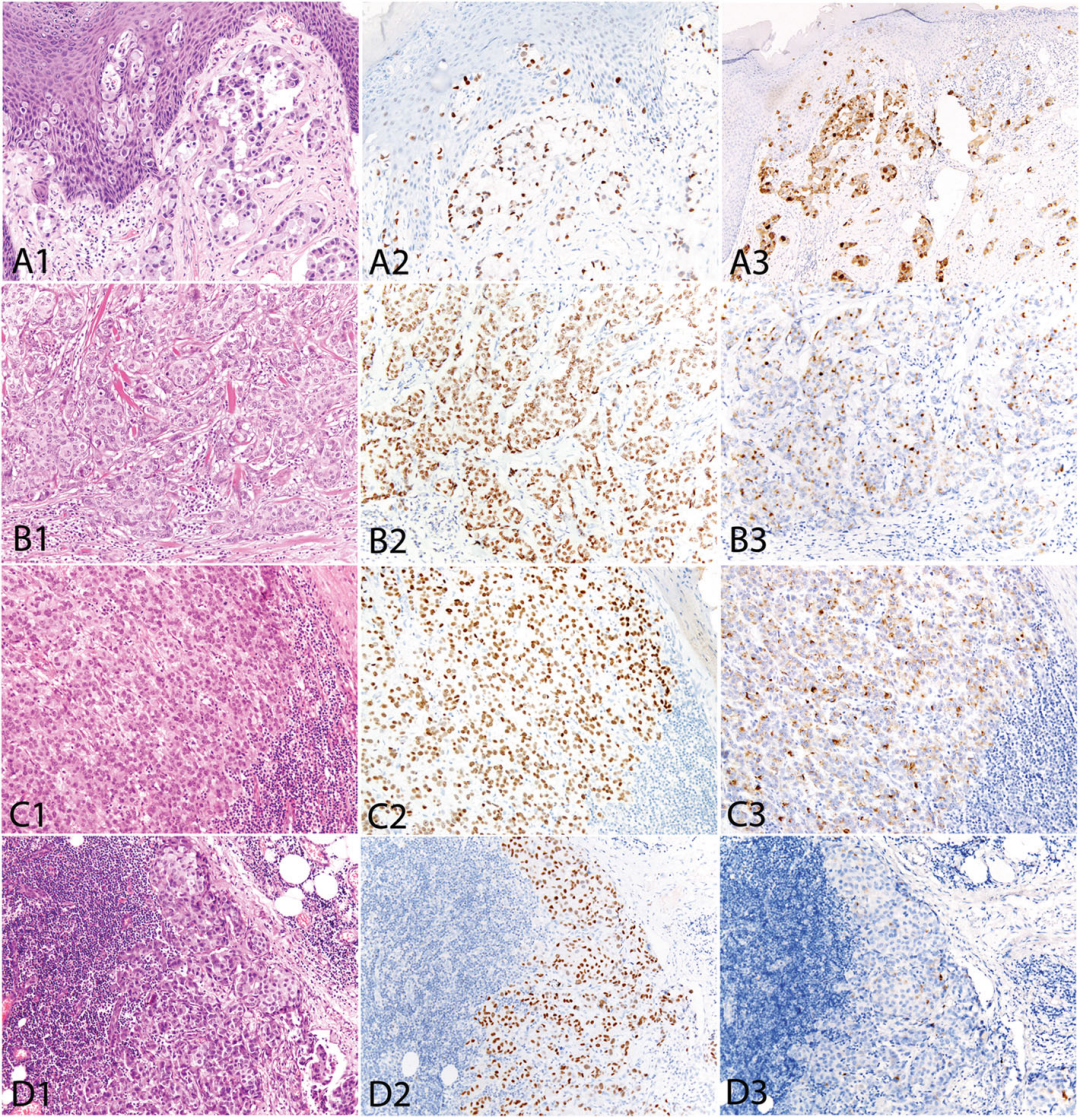
The invasive adenocarcinoma in type Ib disease (primary extramammary Paget disease with invasive adenocarcinoma) (A1: vulva, with intracytoplasmic mucin; B1: scrotum) showed positive GATA3 staining in all but one cases (A2, B2) (A2 also with intraepithelial disease). Most of such invasive adenocarcinomas were also positive for GCDFP15 staining (A3, B3). All 3 metastatic adenocarcinomas from vulvar (C1) and 11 of 12 metastatic adenocarcinomas from penoscrotal (D1) extramammary Paget diseases were positive for GATA3 (C2, D2) and most of them were also positive for GCDFP15 (C3, D3). GATA3 stains more tumor cells than GCDFP15 in some cases (D2, D3)

**Fig. 3:**
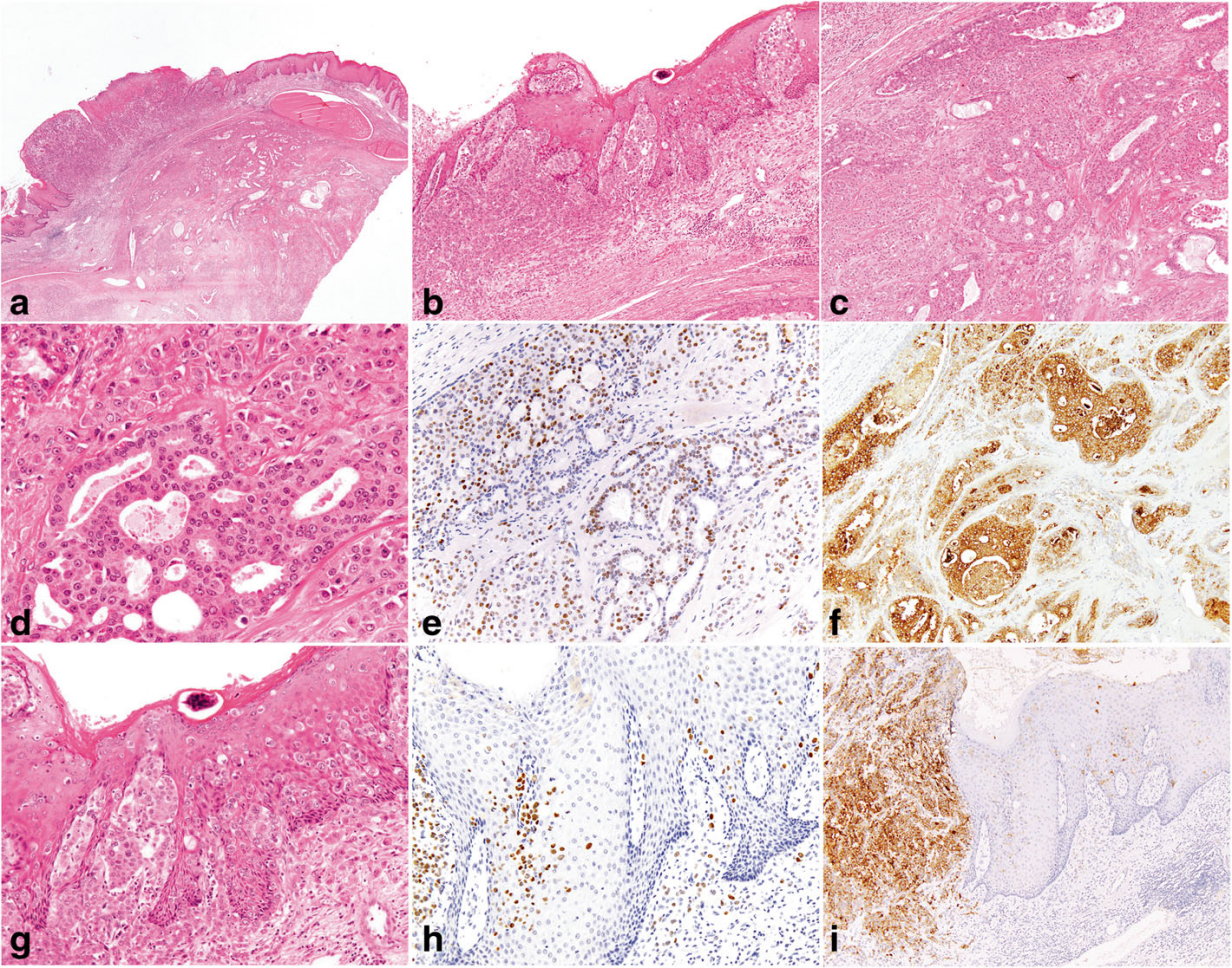
Immunohistochemical staining of GATA3 and GCDFP15 in a vulvar primary extra-mammary Paget disease with an underlying apocrine adenocarcinoma (type Ic disease). The underlying apocrine adenocarcinoma formed a mass in the dermis and subcutaneous tissue but it eroded the epidermis (**a**) and grew in a pagetoid pattern within the adjacent epidermis (**b**, **g**). The underlying adenocarcinoma showed nests, solid and glandular growth patterns with some cribriform glands (**c**, **d**). Cytoplasmic apical apocrine snouts were apparent (**d**). Both the underlying invasive apocrine adenocarcinoma (**d**) and the overlying intraepithelial Paget disease (**g**) were positive for GATA3 (**e**, **h**) and GCDFP15 (**f**, **i**). Similar GATA3 findings were also observed in penoscrotal type Ic diseases

All 34 intraepithelial disease components showed positive GATA3 staining (34/34, 100%), including 1+ in 2 (6%), 2+ in 1 (3%), 3+ in 3 (9%) and 4+ in 28 (82%), with almost all cases demonstrating moderate to strong nuclear staining (Fig. 1). The invasive adenocarcinoma showed positive GATA3 staining in all 10 cases (2+ in 2, 4+ in 8) (Figs. 2, 3). All 3 metastatic adenocarcinomas from vulvar EMPDs showed 4+ GATA3 staining (Fig. 2).

Positive GCDFP15 staining was seen in 28 of 34 (82%) intraepithelial disease components (1+ in 12/28, 2+ in 4/28, 3+ in 4/28, 4+ in 8/28) and 9 of 10 (90%) invasive adenocarcinomas (1+ in 3/10, 3+ in 1/10, and 4+ in 5/10; 5/5 type Ib disease, 4/5 type Ic disease) (Table 1, Figs. 2, 3). The only invasive adenocarcinoma that was negative for GCDFP 15 staining was the eccrine carcinoma (type Ic disease). Two of the 3 (66%) metastatic adenocarcinomas showed positive GCDFP15 staining (1+, 4+) (Fig. 2).

### Expression of GATA3 and GCDFP15 in male primary extramammary Paget diseases

Among the 37 cases of male extra-mammary Paget diseases, 33 were from the scrotum, 3 from the penis and 1 from the perineum. Twenty-one (57%) had intraepithelial disease only. The remaining 16 cases (16/37 or 43%) had both intraepithelial disease and invasive adenocarcinoma (14 type Ib, 2 type Ic) including 12 (12/16, 80%) with lymph node metastases. The staining results of GATA3 and GCDFP15 for each component of male extra-mammary diseases are summarized in Table 2.

**Table 2:** Immunohistochemical staining results of GATA3 and GCDFP15 in primary male genital extramammary Paget diseases

Disease Component	GATA3 staining^a^						GCDFP15 staining^a^						*P* value
	0	1+	2+	3+	4+	Total	0	1+	2+	3+	4+	Total	
Intraepithelial disease (*N* = 37)	0	4 (11%)	2 (6%)	2 (6%)	29 (78%)	37 (100%)	19 (51%)	12 (32%)	4 (11%)	0	2 (6%)	18 (49%)	< 0.001
Type Ia (*N* = 21)		1			20		17	4					
Type Ib (*N* = 14)		3	1	2	8		1	7	4		2		
Type Ic (*N* = 2)			1		1		1	1					
Invasive adenocarcinoma (*N* = 16)	1 (6%)	2 (13%)	2 (13%)	1 (6%)	10 (63%)	15 (94%)	5 (31%)	6 (38%)	1 (6%)	0	4 (25%)	11 (69%)	0.062
type Ib (*N* = 14)	1	2	1	1	9		5	5			4		
type Ic (*N* = 2)			1		1			1	1				
Metastatic adenocarcinoma (*N* = 12)	1 (8%)	1 (8%)	1 (8%)	1 (8%)	8 (67%)	11 (92%)	2 (17%)	3 (25%)	2 (17%)	1 (8%)	4 (33%)	10 (83%)	0.277

^a^The staining is semi-quantitatively scored as follows: 0: < 1% tumor cell staining; 1+: 1–25% tumor cells staining; 2+: 26–50% tumor cells staining; 3+: 51–75% tumor cells staining; 4+: 76–100% tumor cells staining

Positive GATA3 staining was seen in all 37 intra-epithelial disease components (100%), including 1+ in 4 (11%), 2+ in 2 (6%), 3+ in 2 (6%), and 4+ in 29 (78%) (Fig. 1). The invasive adenocarcinomas showed positive GATA3 staining in 15/16 (94%) cases including 1+ in 2 (12%), 2+ in 2 (12%), 3+ in 1 (6%) and 4+ in 10 (63%) (Fig. 2). The invasive adenocarcinoma in type Ib disease was positive for GATA3 in 13/14 cases (93%). The underlying invasive adenocarcinomas in 2 type Ic EMPDs were both positive for GATA3 (2+, 4+). Positive GATA3 staining was seen in 11/12 (92%) metastatic adenocarcinomas, including 1+ in 1 (8%), 2+ in 1 (8%), 3+ in 1 (8%) and 4+ in 8 cases (67%) (Fig. 2).

Positive GCDFP15 staining was seen in 18 of 37 (51%) intraepithelial disease components including 1+ in 12 (32%), 2+ in 4 (11%), and 4+ in 2 (6%) (Fig. 1). The invasive adenocarcinomas showed positive GCDFP15 staining in 11 of 16 (69%) cases including 1+ in 6 (38%), 2+ in 1 (6%) and 4+ in 4 (25%). The invasive adenocarcinoma in Type Ib disease was positive for GCDFP15 in 9/14 (64%, 1+ in 5/9, 4+ in 4/9) cases. The 2 invasive adenocarcinomas in type IC EMPDs showed positive GCDFP15 staining in both (1+, 2+). Positive GCDFP15 was seen in 10 of 12 (83%) metastatic adenocarcinomas (1+ in 3/12, 2+ in 2/12, 3+ in 1/12, 4+ in 4/12) (Fig. 2).

### Comparison of GATA3 to GCDFP15 in primary genital extramammary Paget diseases

Among the 71 intraepithelial diseases (34 from female, 37 from male), all (100%) showed GATA3 staining whereas only 46 of them (46/71 or 65%, female 28/34 or 82%, male 18/37 or 51%) showed positive GCDFP15 staining (*p* < 0.0001). Among the invasive adenocarcinomas, positive GATA3 and GCDFP15 staining was seen in 25/26 (96%) and 18/26 (69%) cases, respectively (*p* = 0.01). Among the 15 metastatic adenocarcinomas, 14 (93%) showed positive GATA3 staining and 12 (80%) showed positive GCDFP15 staining (*p* = 0.2825).

Among the cases with positive immunohistochemical staining, the mean percentage of positively stained tumor cells in the intraepithelial diseases was 79% (female 83%, male 76%) for GATA3 and it was 25% (female 35%, male 10%) for GCDFP15 (*p* < 0.001). As far as the invasive adenocarcinomas were concerned, the mean percentage of tumor cells positive for GATA3 and GCDFP15 was 71% (female 76%, male 68%) and 34% (female 42%, male 34%), respectively (*p* < 0.001). The mean percentage of GATA3-positive metastatic adenocarcinoma cells was 73% (female 90%, male 68%) and it was 50% for GCDFP15 (female 65%, male 48%) (*p* < 0.01).

### Expression of GATA3 in normal epidermal cells

Positive GATA3 staining was seen in some normal epidermal cells in 22/34 (65%) female and 22/37 (60%) cases, respectively, mainly in the spinous layer (typically focal but occasionally diffuse) with occasionally in the basal layer. Among the GATA3 positive cases, the GATA3 staining intensity in the normal epidermal cells was weaker than that in PD in 16/22 (73%) female and 17/22 (77%) cases, respectively. Similar GATA3 staining intensity was seen in the normal epidermal cells and in the intraepithelial PD cells in 6 of 22 (27%) female and 5 of 22 (23%) male cases, respectively. The intraepithelial PD cells typically have larger nuclei than normal epidermal cells. However, in 2 cases in each gender, the intraepithelial PD cells focally have small nuclei and in these areas it is difficult to distinguish the intraepithelial PD cells from normal epidermal cells just based on immunohistochemical staining. Their distinction relies on the growth pattern.

## Discussion

In this study, we investigated the immunohistochemical expression of GATA3 in a large series of 72 primary EMPDs in male and female genital regions. We found that GATA3 was highly expressed in the primary genital EMPDs. The high sensitivity of GATA3 is not only present in the intraepithelial disease (100%) but also in the invasive adenocarcinomas (96%) and metastatic adenocarcinomas (93%). These results indicate that GATA3 is a very sensitive marker for primary EMPDs in the genital regions.

GATA3 is a zinc-finger transcription factor involved in embryogenesis, cell proliferation and differentiation in multiple human tissues and organs, including breast, genitourinary system, parathyroid, skin, central nervous and hematopoietic systems [33–36]. In 2007, Higgins et al. found that GATA3 was a sensitive diagnostic marker for urothelial carcinoma [37]. Since then, there has been growing evidence that GATA3 could serve as a relatively sensitive diagnostic marker for breast carcinomas, parathyroid tumors, trophoblastic tumors, mesonephric adenocarcinomas, paragangliomas and pheochromocytomas etc. [28–31, 38–42]. Other tumors with a less frequent expression of GATA3 include salivary gland tumors, malignant mesotheliomas, pancreatic adenocarcinomas, skin squamous cell carcinomas, skin adnexal tumors, renal oncocytomas, chromophobe renal cell carcinomas, and yolk sac tumors [28–30]. Morbeck D et al. recently reported positive GATA3 expression in all 11 vulvar primary EMPDs (4 with invasive carcinoma) [32]. They did not include any metastatic adenocarcinoma from vulvar Paget disease. They did not study male genital EMPDs, either. Our findings and that of Morbeck et al. [32] add primary genital EMPDs to the list of tumors with ***high*** expression of GATA3. High expression of GATA3 in EMPDs has some diagnostic implications, both for primary EMPDs and their metastasis.

Distinguishing primary from secondary EMPDs is clinically critical given their different treatment and prognosis [3, 9]. Secondary EMPD in the genital region is usually the result of intraepithelial spread from a visceral carcinoma, with urogenital tract (urothelial carcinoma, prostate) and the gastrointestinal tract (distal colon, rectum) being the most 2 common sources [1–3, 6, 9, 12–16]. In females, secondary EMPD in vulva caused by urothelial carcinoma typically involves periurethral vulvar vestibule but it may extend to the adjacent vulvar skin and it may also become invasive [1–3, 6, 9, 12–16]. In males, both urothelial carcinoma and prostate carcinoma may involve scrotum in an intraepithelial pagetoid fashion [43–45]. Rarely urothelial carcinoma [43, 46] and prostate adenocarcinoma [43, 47, 48] may recur in the penis as a secondary EMPD. Since both primary EMPDs and urothelial carcinomas are positive for GATA3, GAT3 is not useful in distinguishing primary EMPDs from secondary EMPD caused by urothelial carcinoma and other markers should be sought for this purpose. Urothelial carcinomas are often positive for uroplakin-III, p63 and p40 whereas EMPDs have an opposite immunohistochemical profile [49–51]. GCDFP15 is often positive in primary EMPDs [20–26] but it is only rarely positive in urothelial carcinoma [43, 52]. Secondary EMPD caused by prostatic adenocarcinoma can be distinguished from primary EMPD by p501S (prostein) and GATA3. Prostatic adenocarcinoma is positive for p501S but negative for GATA3 whereas EMPD shows an opposite profile [28–30, 43, 53]. Prostatic adenocarcinoma can be rarely positive for GCDFP15 and primary EMPDs can show positive prostate specific antigen (PSA) staining in as many as 30% cases [43, 53]. Therefore, one cannot rely on PSA or GCDFP15 to distinguish primary EMPD from secondary PD caused by prostatic adenocarcinoma. Secondary EMPDs from anorectal adenocarcinomas typically extend from perianal skin to the vulva or scrotum [1–6, 9, 43, 44]. GATA3 is negative in colorectal adenocarcinoma [28–30] and therefore is useful to distinguish primary genital EMPD from secondary EMPD due to colorectal adenocarcinoma. It should be pointed out that primary EMPDs can be rarely positive for CDX2 (3%) [43]. GCDFP15 is negative in colorectal adenocarcinomas [43]. Anorectal adenocarcinoma and primary vulvar EMPDs showed overlapping profiles in CK7 and CK20 though CK7 negativity favors the former and CK20 negativity favors the latter [43]. In the genital area, rare pagetoid squamous cell carcinoma in situ can closely mimic intraepithelial EMPD [54, 55] and may be misdiagnosed as such [54]. Since some squamous cell carcinomas and normal epidermal cells are positive for GATA3 [28–30], GATA3 is not useful to distinguish primary EMPD from pagetoid squamous cell carcinoma in situ. Instead P63 should be used in this scenario (p63 negative in primary EMPD but positive in pagetoid squamous cell carcinoma in situ) [50, 51]. Lastly, melanoma in situ may closely mimic primary EMPD and rare pigmented primary EMPD has been reported [56, 57]. Melanoma in situ was negative for GATA3 [28–30] but positive for S100, melan-A and HMB45 whereas EMPD had an opposite immunoprofile.

Although most primary EMPDs are intraepithelial, approximately 4% to 20% primary vulvar EMPDs [1–4, 9, 13, 15, 16] and 26% to 61% primary penoscrotal EMPDs were invasive at the time of presentation [5, 7, 8, 10, 58–61]. Some of these invasive adenocarcinomas arise from the intraepithelial EMPD (type Ib primary EMPDs) whereas others are underlying adenocarcinomas which showed secondary epidermotropism (type Ic primary EMPDs) [3, 6, 8, 9, 14–16]. In vulva, it is estimated that type Ic EMPDs account for at least 10–30% invasive EMPDs [1–3, 8, 9, 15, 16, 62]. Rare type Ic primary EMPD in penoscrotum has also been reported [63] and two of our cases belong to this category. Type Ic EMPDs were reported to be associated with a worse prognosis than type Ib EMPDs [3, 9] and therefore pathologists should attempt to specify the subtypes of primary invasive EMPDs (Ib versus Ic). However, it is not always feasible to distinguish them. Our findings indicate that type Ib and apocrine type Ic diseases cannot be distinguished by their GATA3 and GCDFP15 immunoprofile given their similar profile for these two markers. Type Ic EMPDs are predominantly of apocrine type, but other types of adenocarcinomas may also rarely give rise to type Ic EMPDs including eccrine sweat gland adenocarcinoma [64], Bartholin gland adenocarcinoma [65], and adenocarcinomas of mammary-like glands [66, 67] etc. One of the invasive adenocarcinomas in vulvar type Ic EMPDs in our study was an eccrine carcinoma. Cutaneous eccrine carcinomas were positive for GATA3 in 36% to 68% cases [68, 69]. The only eccrine carcinoma in our study showed 4+ GATA3 staining (>75% cells). Thus, GATA3 immunostaining cannot distinguish type Ib EMPDs from apocrine and *eccrine* type Ic primary EMPDs. Their distinction relies on morphology and other markers such as p63 and GCDFP15. Eccrine carcinomas were often positive p63 (85% to 89%) whereas primary type Ib EMPDs were not [68, 69]. Eccrine carcinomas were only rarely positive for GCDFP15 (5%) [69]. Adenocarcinoma of mammary-like gland in the vulva is rare and its diagnosis requires the presence of a transition zone between normal mammary-like glands and adenocarcinoma [66, 67, 70]. Morphologically it is similar to breast carcinoma. Both ductal type [66] and lobular-like [67] mammary-like carcinomas with Paget’s disease (type Ic primary EMPD) have been reported. Although there has been no report of GATA3 in vulvar adenocarcinoma of mammary-like glands, it is conceivable that the vast majority of these tumors will be positive for GATA3 as in breast carcinoma. As expected, two thirds of vulvar mammary-like carcinomas were also positive for GCDFP15 [70]. For these reasons, rare type IC primary EMPD due to mammary-like carcinoma cannot be distinguished from type Ib EMPD or type IC EMPD due to sweat gland adenocarcinoma by GATA3 and GCDFP15 immunostaining. Primary type Ic EMPDs caused by underlying apocrine carcinomas were often negative for ER and PR. In contrast, vulvar mammary-like carcinomas were often positive for these two markers [66, 67, 70–72].

Among patients with invasive EMPDs (type Ib and Ic), some will develop metastatic disease at the time of presentation or in their subsequent disease courses. In the SEER data, 17.1% patients with invasive EMPDs have lymph node metastasis (male 16.0%, female 17.6%) and 2.5% have distant metastasis (male 3.8%, female 1.9%) at presentation [4]. In a recent Japanese study of 301 primary invasive EMPDs (both male and female), 114 (37%) had metastasis including 20% node metastasis and 17% distant metastasis (16% with both nodal and distant metastasis) [12]. Lymph nodes metastasis typically involved inguinofemoral nodes but pelvic and para-aortic nodes were also involved in some patients [9–11, 22, 58–62]. Distant metastatic sites include bone, lung, liver, lung, brain and muscle [9, 58–62]. Invasive EMPDs were morphologically similar to other types of tumors especially breast carcinoma and urothelial carcinoma, and therefore they may pose some diagnostic difficulty in metastasis, which can be further complicated by the fact that patients with primary EMPDs have an increased risk of developing other types of secondary primary tumors (overall 5–8% chance). Breast carcinoma and urothelial carcinoma are among the most common secondary tumors in these patients, and they can occur either before or after the diagnosis of primary EMPDs [6, 9, 12, 14–17, 58–60, 62]. In patients with both a primary invasive EMPD and another type of tumor (particularly urothelial and breast carcinoma), the differential diagnosis for a metastatic tumor with positive GATA3 staining should also include metastatic primary EMPDs in the list of differential diagnosis. A panel of immunohistochemical markers should be used to facilitate the correct diagnosis.

GCDFP15 was a useful marker for primary EMPDs but its sensitivity was 60% to 85% [20–26]. In this study, we showed that GATA3 is relatively more sensitive than GCDFP15 for primary EMPDs, especially in male patients. Our study is the largest series of primary EMPDs with GCDFP15 staining. It is interesting to note that GCDFP15 stains a higher percentage of primary EMPDs in female patients than male patients.

Although GATA3 is a sensitive marker for primary genital EMPDs, it should be pointed out that it is not specific for these tumors. As described above and reviewed elsewhere, several other types of tumors including urothelial carcinoma, breast carcinoma, paragangliomas/pheochromocytomas, trophoblastic tumors, and mesonephric adenocarcinomas are often positive for GATA3 [28–31, 38–42]. In this sense, GATA3 is less specific than GCDFP15 for primary genital EMPDs. In difficult cases particularly in metastasis, both GATA3 and GCDFP15 should be used in conjunction to avoid misdiagnosis.

Lastly, high expression of GATA3 in primary EMPDs may also help shed some lights on the histogenesis of these tumors. Currently there are 3 theories: intraepidermal origin of adnexal origin such as apocrine glands, multipotent stem cells in the epidermis or infundibular stem cells from hair follicles [1, 2, 20, 73]. Positive staining for both GATA3 and GCDFP15 in primary EMPDs probably favors the first theory.

One limitation of our study is that we did not include genital secondary EMPDs. Secondary EMPDs are rare and it is difficult to collect a meaningful number of cases to do a comparison study. The two most common types of carcinomas that cause secondary EMPDs are urothelial carcinoma and colorectal carcinoma [1–3, 9, 13–17]. As discussed above, GATA3 immunoreactivity was seen in most urothelial carcinomas but not in colorectal carcinomas [28–30, 37].

## Conclusions

In summary, we investigated immunohistochemical expression of GATA3 in a large series of 72 primary EMPDs in the male and female genital regions. Our findings show that GATA3 is a very sensitive marker for genital primary EMPDs and is more sensitive than GCDFP15. Although GATA3 is highly sensitive for primary EMPDs, it is not specific for these tumors. GATA3 staining cannot distinguish intraepithelial PD from pagetoid squamous cell carcinoma in situ or primary EMPD from secondary EMPD caused by urothelial carcinoma. GATA3 staining can be used to distinguish primary EMPD from pagetoid melanoma in situ and secondary EMPD caused by colorectal carcinoma. In the metastatic setting, GATA3-positive tumors should include metastatic adenocarcinoma originated from PD.

## Abbreviations

EMPD: Extramammary Paget disease
GATA3: GATA-binding protein 3
GCDFP15: Gross Cystic Disease Fluid Protein 15
PD: Paget disease
